# Automated measurement of cell mechanical properties using an integrated dielectrophoretic microfluidic device

**DOI:** 10.1016/j.isci.2022.104275

**Published:** 2022-04-20

**Authors:** Hao Yang, Mingjie Zhu, Tao Chen, Fuzhou Niu, Lining Sun, Liang Cheng

**Affiliations:** 1 Robotics and Microsystems Center, School of Mechanical and Electric Engineering, Soochow University, Suzhou; 2 School of Mechanical Engineering, Suzhou University of Science and Technology, Suzhou; 3 Institute of Functional Nano & Soft Materials (FUNSOM), Collaborative Innovation Center of Suzhou Nano Science and Technology, Soochow University, Suzhou

**Keywords:** Fluidics, Cell biology, Technical aspects of cell biology

## Abstract

Cell mechanics is closely related to and interacts with cellular functions, which has the potential to be an effective biomarker to indicate disease onset and progression. Although several techniques have been developed for measuring cell mechanical properties, the issues of limited measurement data and biological significance because of complex and labor-intensive manipulation remain to be addressed, especially for the dielectrophoresis-based approach that is difficult to utilize flow measurement techniques. In this work, a dielectrophoresis-based solution is proposed to automatically obtain mass cellular mechanical data by combining a designed microfluidic device integrated the functions of cell capture, dielectrophoretic stretching, and cell release and an automatic control scheme. Experiments using human umbilical vein endothelial cells and breast cells revealed the automation capability of this device. The proposed method provides an effective way to address the low-throughput problem of dielectrophoresis-based cell mechanical property measurements, which enhance the biostatistical significance for cellular mechanism studies.

## Highlights

• Cell capture, dielectrophoretic stretching, and release in one microfluidic chip
• Automatic measurement scheme to realize circularly measurement
• Automatic acquisition of large amounts of cell mechanical properties data
• Significant advances in dielectrophoretic measurement of cell mechanical properties

## Graphical abstract

**Figure:**
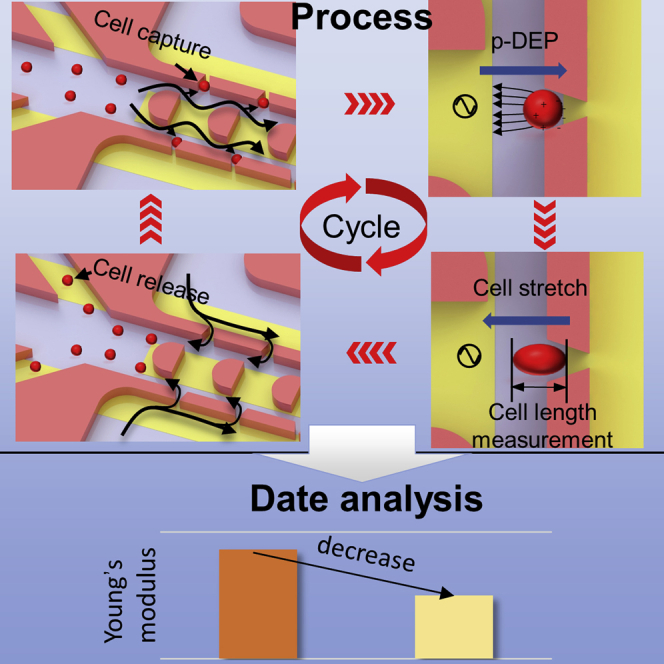

## Introduction

Fundamental cell biological processes, including cell division, differentiation, and metastasis, are affected by mechanical factors (Bausch and Kroy 2006; Nagayama et al., 2001; Sens and Plastino 2015). Cell biomechanics can characterize their physiological functions to a certain extent (Discher et al., 2009). Cells in different states exhibit varying mechanical properties (Barabino et al., 2010; Chien 1985; Li et al., 2017). For example, metastatic cancer cells are softer than normal cells (Cross et al., 2007; Swaminathan et al., 2011). Healthy red blood cells (RBCs) are less stiff than sickly ones (Brandao et al., 2003). Therefore, a quantitative comparison of the deformability of cells in different physiological states could reveal or diagnose a certain disease in advance or evaluate the efficacy of a certain drug (Wang et al., 2013; Zhang et al., 2015).

Methods for measuring cell mechanical properties can be divided into two categories: contact and noncontact. Contact measurements generally utilize micropipette aspiration (Evans and Hochmuth 1976; Hochmuth 2000; Rosenbluth et al., 2006), atomic force microscopy (AFM) (Takahashi et al., 2014), elastic membranes (Zheng et al., 2012), and microfluidic constriction (Lange Janina et al., 2017), etc. Micropipette aspiration sucks the cells into an extremely thin tube with a force ranging from 10 pN to 10 μN to monitor their deformation under different pressure control and then evaluate their elastic modulus (Hochmuth 2000). However, it relies on the operating experience, and the accuracy is not properly measured. AFM is a tool that can directly characterize cell deformation by applying microprobes on cells, which has disadvantages including low throughput, high complexity, and high equipment requirements. The elastic membrane can be used to evaluate the mechanical properties of cells by being seeded with cells, followed by stretching and contraction to observe cellular deformation. This method requires high precision for the stretching equipment and biocompatibility for the elastic membrane. Microfluidic contraction is to make cells flow in long and narrow channels and to measure mechanical properties by comparing parameters such as deformation length and transit time. Although it is possible to obtain large volumes of data, the measured parameters are influenced by the adhesion properties of cells, and the multiparameter measurement cannot be qualitatively compared with other methods.

Noncontact methods mainly include optical tweezers (Tan et al., 2012; Wang et al., 2013), magnetic tweezers (Chen et al., 2015; Kasetsirikul et al., 2016; Mijailovich et al., 2002), hydrodynamic stretching (Gossett et al., 2012), and dielectrophoresis (DEP) (Cen et al., 2004; Chen et al. 2011, 2014; Doh et al., 2012; MacQueen et al., 2010). Optical tweezers use highly focused laser beams to achieve cell fixation and stretching. Nevertheless, the force generated by the optics is weak and thus limits the types of cells that can be detected (Hou et al., 2009). Magnetic tweezers employ magnetic beads to adhere to the cells and then complete the arbitrary distortion of the cells according to the magnetic attraction to detect their mechanical properties. The position deviation of the magnetic beads may result in large measurement errors and low measurement accuracy (Mijailovich et al., 2002). Hydrodynamic stretching is a method for evaluating mechanical properties by deforming cells in a microfluidic channel under the combined action of shear and compressive forces. Flow measurement can effectively achieve high-throughput measurement; however, there are several problems such as potentially-destructive assay and possible channel clogging. DEP is one of the most effective noncontact cell measurement methods to utilize the principle of particle movement under a nonuniform electric field because of the polarization effect. Cell electrode formation based on DEP was first reported in 1984 (Engelhardt et al., 1984). DEP has low requirements for experimental equipment and has the potential to achieve high-throughput cell stretching detection by combining it with other techniques, i.e., automation. Therefore, the DEP-based method is in line with the future direction of cellular mechanical property measurements (Hao et al., 2020). Guido et al. tested the deformation of MCF-7 and MCF-10A to determine the difference in mechanical properties between healthy cells and cancerous cells (Guido et al., 2012). Zhang et al. compared Young’s modulus of drug-treated NB4 cells with untreated cells through DEP to evaluate the drug quality (Zhang et al., 2015). Qiang et al. employed the design of an interdigital electrode array to study the deformability of cyclic stretch-release loaded RBCs (Qiang et al., 2017b). Shalileh et al. conducted a pathological evaluation based on the different characteristics of the DEP response of cancerous cells and noncancerous cells and compared them with existing biological methods (Shahriar Shalileh, et al.,2021).

It can be concluded that the current techniques are capable of measuring cell deformation. However, the DEP-based measurement method still suffered from low measurement efficiency and high manpower requirements. The reason for this is that DEP-based methods require cell capture at the edge of the electrodes before stretching, making it time-consuming and difficult to use existing continuous fluidic operation techniques. Thus, a new way that can measure the mechanical properties of cells in large quantities and with low manpower requirements using dielectrophoresis is highly needed.

This article focuses on the development of a DEP-based high-throughput automated measurement method for cellular mechanical properties, which combines a newly designed integrated multifunctional microfluidic device and an automatic control scheme. The integrated dielectrophoretic microfluidic device mainly includes three functions: array capture at the single-cell level using hydrodynamic to avoid the interaction between cells and reduce spatial clutter, stretching operation of cells using DEP to effectively measure the mechanical properties of cells, and cell release operation to wash away the cells that have been stretched by applying reversed pressure, thus providing reliable support for multi-batch measurement of cell mechanical properties. The automatic control scheme mainly includes an image processing module and hardware control module. The former was used to measure the stretch of the cell and store the cell shape variable in real time, and the latter controlled the signal generator and microfluidic pump logically and felicitously to complete the automated measurement of cells. The dielectrophoretic microfluidic device is combined with the automatic control scheme to obtain large quantities of mechanical characteristic data with low manpower requirements. A series of experiments were performed to verify the reasonable feasibility of the system. Large amounts of biological data were also obtained to compare the mechanical properties of normal human umbilical vein endothelial cells (HUVECs) and TNF-α-treated HUVECs and normal MCF-10A and Cytochalasin B (CB)-treated MCF-10A. The proposed method solves the problems of low data volume and high manpower requirement for measuring the mechanical properties of cells based on dielectrophoresis, which is not only highly reliable but also can automatically measure a large number of cells, laying the foundation for the wide applications of the DEP-based measurement methods.

### Design of the microfluidic device

**Figure 1:**
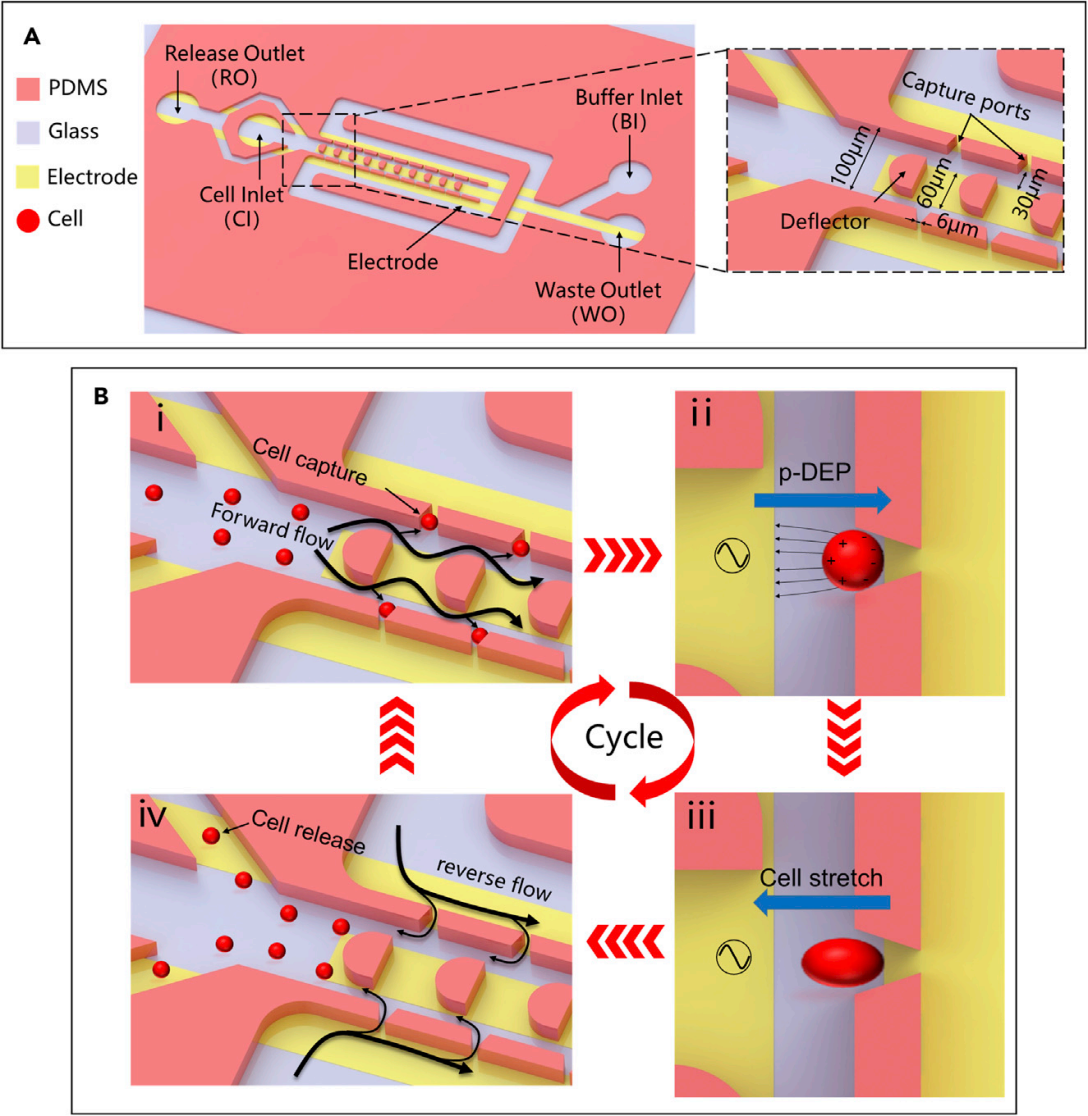
Structure and working principle of the microfluidic device (A) Structure of the entire device without the PDMS cover for enhanced visibility. The close-up shot shows the bonding position between the electrode and the channel and the detailed size design. (B) Working principle and circular working process. First, physical capture of single cells is achieved under the driving force of fluid drag. Second, cell electrical capture and stretching are achieved based on DEP. Third, the cells are released from the capture ports with the reverse flow.

The overall structure design of the integrated dielectrophoretic microfluidic device and the principle for cell capture, stretching, and release are shown in Figure 1. The microfluidic device is composed of a microchannel layer, an indium tin oxide (ITO) electrode layer, and a glass substrate layer. The microchannel consists of the main channel with a width of 100 μm and both bypass channels each with a width of 100 μm (Figure 1A). Deflectors are arranged on the main channel to enhance the probability of cell capture (Luo et al., 2017; White et al., 2011; Yesilkoy et al., 2016). Several capture ports of 6 μm narrow size and 18 μm wide size are placed between the main channel and the bypass channel to capture single cells in fixed positions. The release outlet (RO) is placed on the same side as the cell inlet (CI) to facilitate the reverse release of cells. The height of all channels is 25 μm. In this device structure, 10 capture ports are located on each side of the main channel at a pitch of 100 μm. The design of the number of arrayed capture ports is primarily limited by the need for sufficiently high magnification to obtain sufficiently high accuracy. The microelectrodes are designed to generate a local *p*-DEP force that stretches cells from their immobilization sites. An AC voltage is applied between both ends of the electrodes. Given the design of the electrode structure (enlarged image in Figure 1A), a strong nonuniform electric field is generated at each capture port and polarizes the cells for stretching. The DEP force produces attraction (*p*-DEP) and repulsion (n-DEP) depending on the frequency of the electric field, the related parameters of the cell, and the conductivity of the buffer. Reasonable parameters were selected through subsequent experiments to achieve cell attraction and deformation.

Cell capture-stretch-release is cycled within the device as shown in Figure 1B. In the first step of one cycle, the cells are loaded onto the CI using pump 1 and then flowed into the RO and the main channel. Only a portion of the cells are captured at the capture ports under the hydrodynamic action, and the remaining cell solution flows out of the RO to reduce the flow rate and pressure. The second step of one cycle is to realize the electric capture and stretching of cells by using AC power-generated DEP force. Cell stretch data were recorded automatically using the deep learning-based cell detection algorithm. In the final step of one cycle, the cells are released because the reverse pressure is applied using pump 2 from the buffer inlet (BI) to the RO. The cells can be easily released as long as a large flow rate is set to increase the pressure difference at the cell capture ports. In this way, a whole measurement cycle for mechanical properties is completed, and the high-throughput measurement of the cells can be achieved by automated repeating this cycle several times.

### Experimental setup

**Figure 2:**
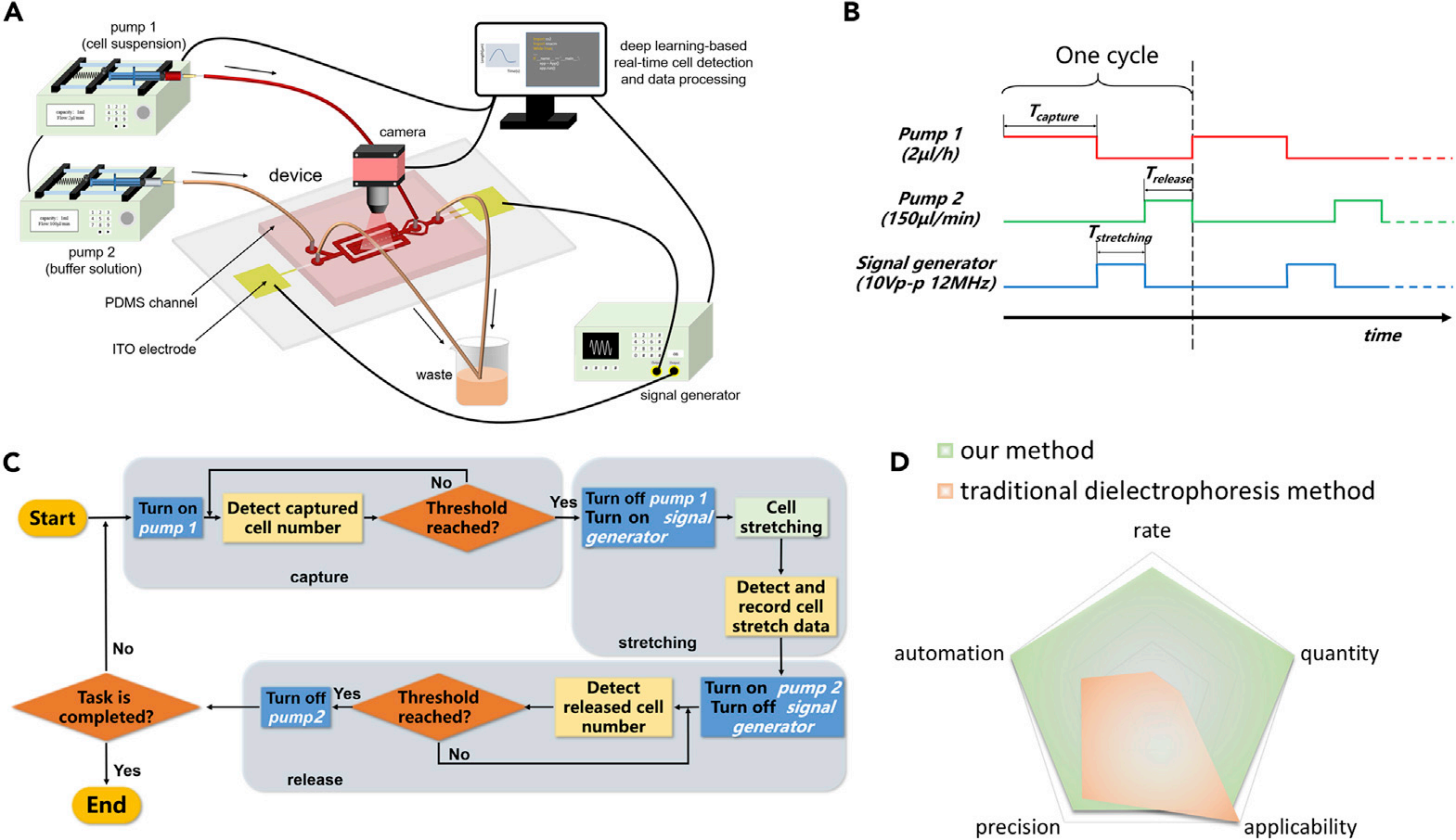
System setup and operation logic (A) The system schematic diagram includes the connection between the microfluidic device and the signal generator and the microfluidic pump and the communication between the hardware and the host computer. The host computer cooperatively controls each component based on visual feedback. (B) The timing diagram of hardware control. (C) Sequence algorithm diagram of a control scheme for the automatic capture-stretch-release of the cells in the device. Different hardware operating states are triggered through the thresholds of cell number and time signal based on image feedback. Hardware operating functions are marked with a blue box in the flowchart. (D) Advantages and disadvantages of the proposed method and the traditional DEP methods

The experimental setup is shown in Figure 2A. An optical microscope (Ti2-U, Nikon, Japan) equipped with a camera (VTSE3S2000, VIHENT, China) was used to observe and record the condition of the cells inside the device. Digital images of 2736 × 1824 pixels (650 × 436 μm under 20× objective lens) were captured at a rate of 50 frames per second. Each image was analyzed in real time to calculate the number of cells captured or released and the feature length of the cells. Both ends of the electrode extending out of the channel were connected to a signal generator (4014B, BK PRECISION, America) using copper wires to provide an AC signal in the range of 0–10 V (peak-to-peak) and a frequency in the range 1 kHz–12 MHz to apply AC electric fields inside the microfluidic cell. The signal generator uses a USB3.0 interface for communication. The CI was connected to pump 1 (LSP02-1B, Halma, U.K) using a 0.5 mm inner diameter polytetrafluoroethylene (PTFE) capillary tube, and the BIwas connected to pump 2. The microfluidic pump uses the RS485 interface to communicate with an industrial computer. A feedback control scheme (implemented in Python) determines the applied voltages and frequencies for the function generator and the flow rate for the microfluidic pump based on the number of cells in specific locations in real time.

The deep learning-based image detection algorithm, which mainly utilizes the framework model of Yolov5, has stronger robustness and generalization ability than traditional image processing methods. Yolov5 employs a separate CNN model to achieve end-to-end target detection, and its network structure includes input, backbone, neck, and prediction. A series of cell images were used to create the training set with annotation tools. The trained weight file was read, and real-time image detection was realized. The algorithm can detect the feature-length and location information of the target and complete the cell eigenvalue detection of a specific region through location screening.

Three system working states referred to as “capture,” “stretch,” and “release” were characterized to realize hardware automatic control based on visual feedback. Each working state corresponds to a different hardware control state as shown in Figure 2B. In the capture states, pump 1 connected to the CI is turned on at a flow rate of 3 μL/h. Owing to the rationality of device design, single cells are gradually captured in a specific position in this step, and an appropriate threshold is set for the number of captured single cells. The threshold is determined by the number of single cells captured in a given period, reducing wasted time and maximizing efficiency. This threshold is the trigger for the second working state. In the stretch states, the signal generator is powered at 10 V voltage and 12 Mhz frequency, and pump 1 is simultaneously turned off. The cells will be stretched because of the action of the nonuniform electric field, and the stretching data will be recorded in real time. This process takes the time threshold as the trigger condition and automatically enters the next working state when it reaches 10 s. In the final release state, only pump 2 connected to the BI is turned on at a flow rate of 150 μL/min to release the detected cells; the number of cells was also used as the trigger threshold (Figure 2C). These three steps comprise a cycle of cell detection. When the detection is finished, the system automatically jumps to the next cycle.

Compared with the traditional DEP method, the proposed method has several advantages. First, the measurement rate is high. The proposed method can measure data batch by batch automatically, and the average measurement rate was about 10 cells (one batch) per minute. In contrast, the traditional DEP method requires a series of separate processes such as video recording, manual selection, manual measurement, etc. After accumulating the time of all the measurement processes, the measurement data per minute is less than 1 (Li et al., 2019). Second, the automatic measurement method we proposed can obtain effective measurement data of more than 1000 cells in one experiment, compared to less than 100 for traditional DEP methods (Du et al., 2014), which is an order of magnitude difference. Third, by adopting the recognition strategy of artificial intelligence, the proposed method is more accurate than the traditional manual measurement method. Fourth, our approach is highly automated and greatly reduces human requirements, whereas the traditional DEP method uses image recognition software for the automatic measurement of cells at most (Qiang et al., 2017a). Fifth, by designing microfluidic channels of different sizes, our method can achieve the same range of applicable cell types as the traditional method. The comparison is shown qualitatively in Figure 2D.

### Fluid and pressure simulation

**Figure 3:**
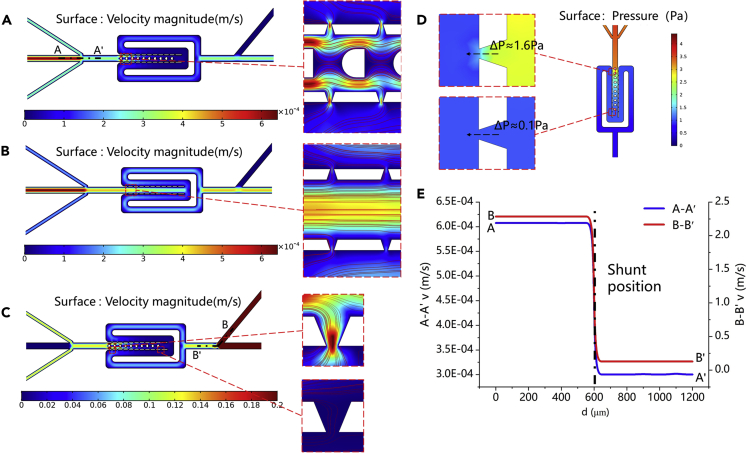
CFD analysis with resulting flow-velocity and pressure (A and B) Distribution of velocity field in the entire area and the streamline distribution in the local enlarged area applying a flow rate at the CI with or without deflectors. (C) Streamline distribution at the first and last positions applying a flow rate at the BI. (D) Pressure simulation distribution map of the entire area, both close-up images show the pressure difference before and after the capture area at the first and last positions, respectively. (E) Diversion situation at AA′ and BB′ cut-off position.

Computational fluid dynamics (CFD) simulation based on the geometric design of this device was first performed to estimate the efficiency of the cell capture process. Figures 3A and 3B illustrate the simulation results of the flow-velocity field; the enlarged image shows the streamlined distribution of the main channel to evaluate the influence of deflectors on cell capture efficiency. Many streamlines are close to the capture ports with deflectors, thereby guiding cells into the capture ports. However, the cells are widely distributed in the main channel without deflectors, and those in the center of the channel may not be captured in time. The cells pass through the capture port driven by the fluid. Given the smaller size of the narrow port compared with that of the cell, the latter is stuck at the capture port under the fluid force. As long as the cell is captured, the capture port is blocked to form an open circuit state, resulting in other cells flowing to other designed capture ports. Figure 3C shows the fluid distribution during the reverse release of cells (a flow rate of 150 μL/min), and the enlarged view illustrates the streamlined distribution in the first and last capture port area. The fluid will pass through all capture ports, even the last one, to ensure that the cells can be easily released from the capture port at a proper flow rate. Surface pressure simulation revealed that the pressure difference between the first and last capture ports ranges from 1.6 Pa to 0.1 Pa at a flow rate of 3 μL/h, indicating that the captured cells are not subjected to huge mechanical damage and will not be squeezed out of the capture port (Figure 3D) (Jin et al., 2015; Luo et al., 2017). Figure 3E shows the velocity distribution at the AA′ section in Figure 3A and the BB′ section in Figure 3C. When the cells are loaded into the device, approximately half of the flow is diverted to the RO. As a result, the flow rate of cells entering the capture area is lowered to reduce the possibility that cells break through the capture port because of excessive pressure and to ensure the integrity of the cell structure. During cell release, the fluid resistance between the waste outlet (WO) and the BI is small because of their close distance. Therefore, only approximately 1/10 of the flow enters the capture area by applying pressure in the BI. The cell release experiment was completed by increasing the flow at the BI without reducing the service life of the device.

## Results and discussion

### Characterization of the capture and release performances

PS beads with 15 μm diameter, which is similar size to cells, were used to characterize the cell capture and release efficiency of microchips with different sizes of the narrow-mouth capture. Based on the pressure simulation, the flow rate of CI was set to 3 μL/h to prevent the cells from being destroyed.

**Figure 4:**
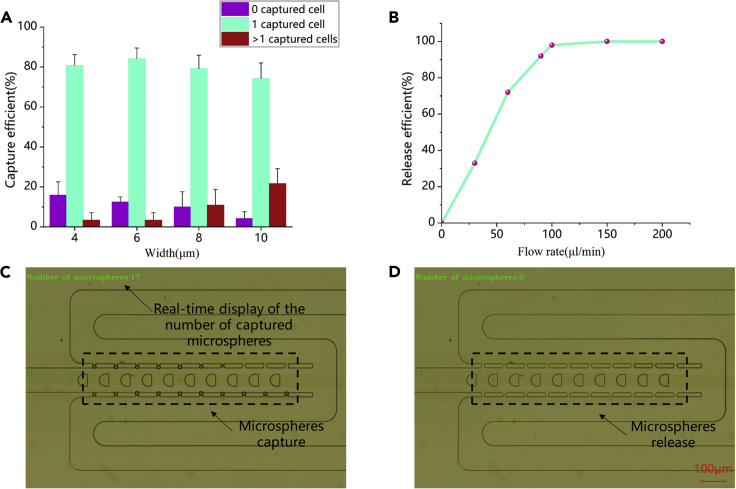
PS bead experiment and automated verification. Data are represented as mean ± SD (A) Capture efficiency of no cell, single-cell, and multiple cells under the narrow-mouth size of 4, 6, 8, and 10 μm. (B) Cell release efficiency as a function of the flow rate. (C and D) Capture and release of PS beads during automatic image recognition; the upper left corner shows the number of real-time PS beads in the capture area. The scale bar denotes 100 μm.

Differently sized (4, 6, 8, and 10 μm) narrow-mouth microfluidic devices were used to compare the capture efficiency of PS beads. Six cycles in a characterization experiment were performed on the same device. Capture efficiency was defined as the ratio of the number of capture ports with a single cell to the total number of capture ports. Figure 4A shows the capture efficiency data. A narrow mouth is associated with a high probability of not capturing PS beads and a low probability of capturing multiple PS beads. The capture efficiency of a single PS bead can reach 84.2% ± 5.3% at 6 μm narrow-mouth size. Given the requirement of maximum capture efficiency of single cells, a device with a narrow-mouth size of 6 μm was selected.

Release efficiency can be optimized by adjusting the flow rate of the BI. This parameter represents the ratio of the number of capture ports that successfully released a single cell to the number of capture ports containing single cells. When the flow rate increases to a certain value, the captured cells rush out to the release outlet under the fluid drag force. PS beads were used to verify the release effect of the device, and Figure 4B illustrates the relationship between the release efficiency and the flow rate. Almost all the PS beads can be released at the flow rate of 100 μL/min. Given the high release efficiency and the service life of the device, a 150 μL/min flow rate was chosen. Figures 4C and 4D display the effects of capturing and releasing PS beads under microscope image detection, in which the upper left corner shows the number of single cells captured in a specific capture area displayed in real-time.

### Mechanical properties difference between normal and drug-treated cells

**Figure 5:**
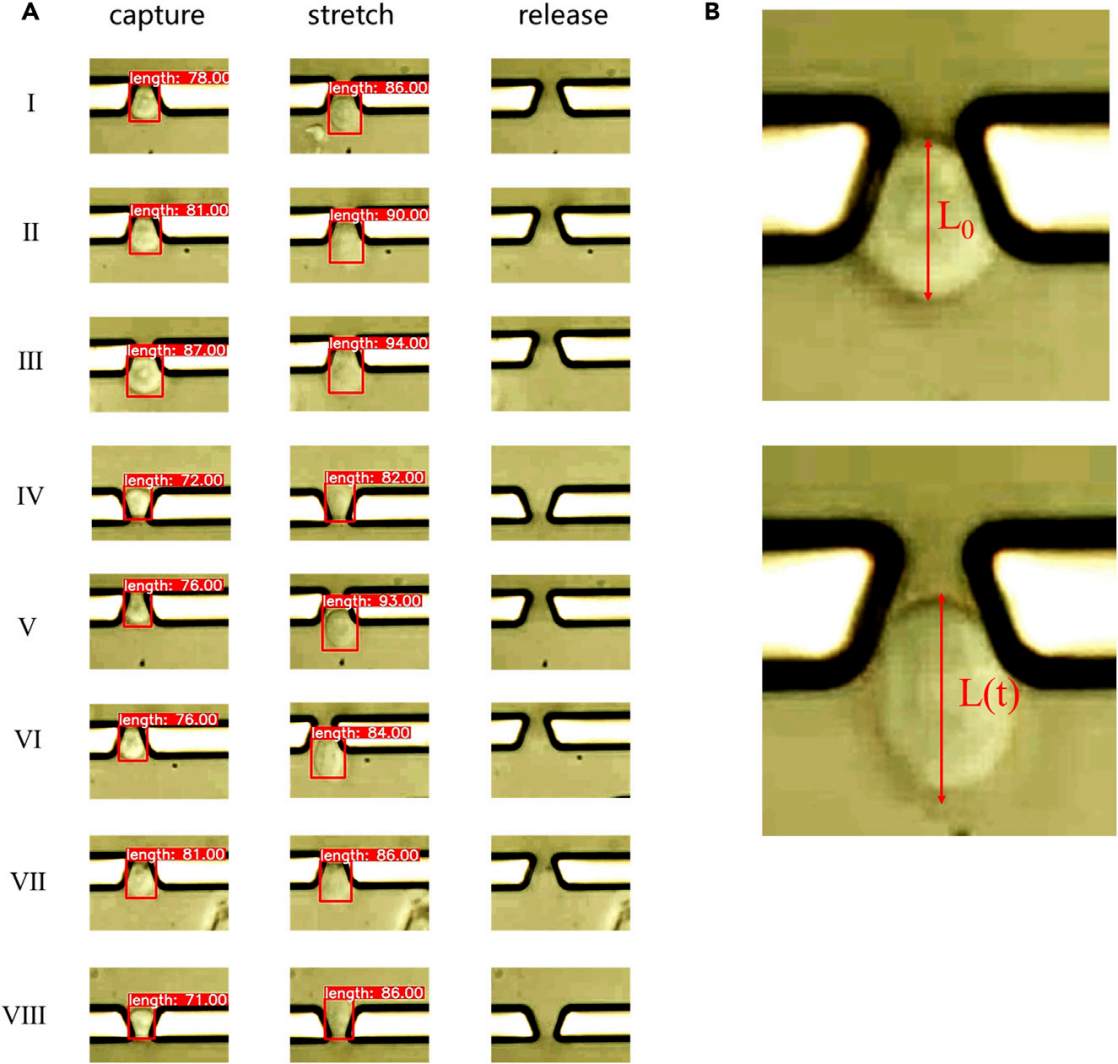
Automated cell experiment (A) Capture, stretch, and release images of cells in the same batch, the length in the red box represents the pixel value of the cell length recognized by Yolov5. (B) The length of the cell before and after stretching.

Figure 5A shows the captured, stretched, and released results of eight relatively complete cells in the same batch of cells within the range of 14 capture ports that can be observed by a microscope. The fluid conditions for the PS bead experiment were also used for the cell experiment. Nevertheless, a small number of cells will still be broken because of the difference in stiffness. The electrode was stimulated by applying an AC voltage to generate *p*-DEP force to stretch the cells in a fixed position. The electrical signal and solution conductivity parameters were set as follows: conductivity of 50 μS/cm, frequency of 12 Mhz, and voltage of 10 Vp-p. These experiments proved the feasibility and effectiveness of capturing, stretching, and releasing cells.

Mass measurement was conducted on the stretch data of normal and TNF-α-treated HUVECs by combining the automated algorithm with the integrated device. TNF-α is a tumor necrosis factor and an inflammatory cytokine that can increase the permeability of vascular endothelial cells and changes in the mechanical properties of cells from the perspective of TNF-α strain on endothelial cells (Chen et al., 2020; He et al., 2020). The strain was defined as ε:

(Equation 1) $$\;\varepsilon \left(t\right)=\frac{L\left(t\right)-L_{0}}{L_{0}},$$

where *L*_*0*_ represents the initial length of the cell, and *L(t)* represents the real-time stretched length of the cell in Figure 5B. When calculating the final strain value, the minimum and maximum lengths of the detected cells were taken as the initial length and the final stretched length, respectively.

**Figure 6:**
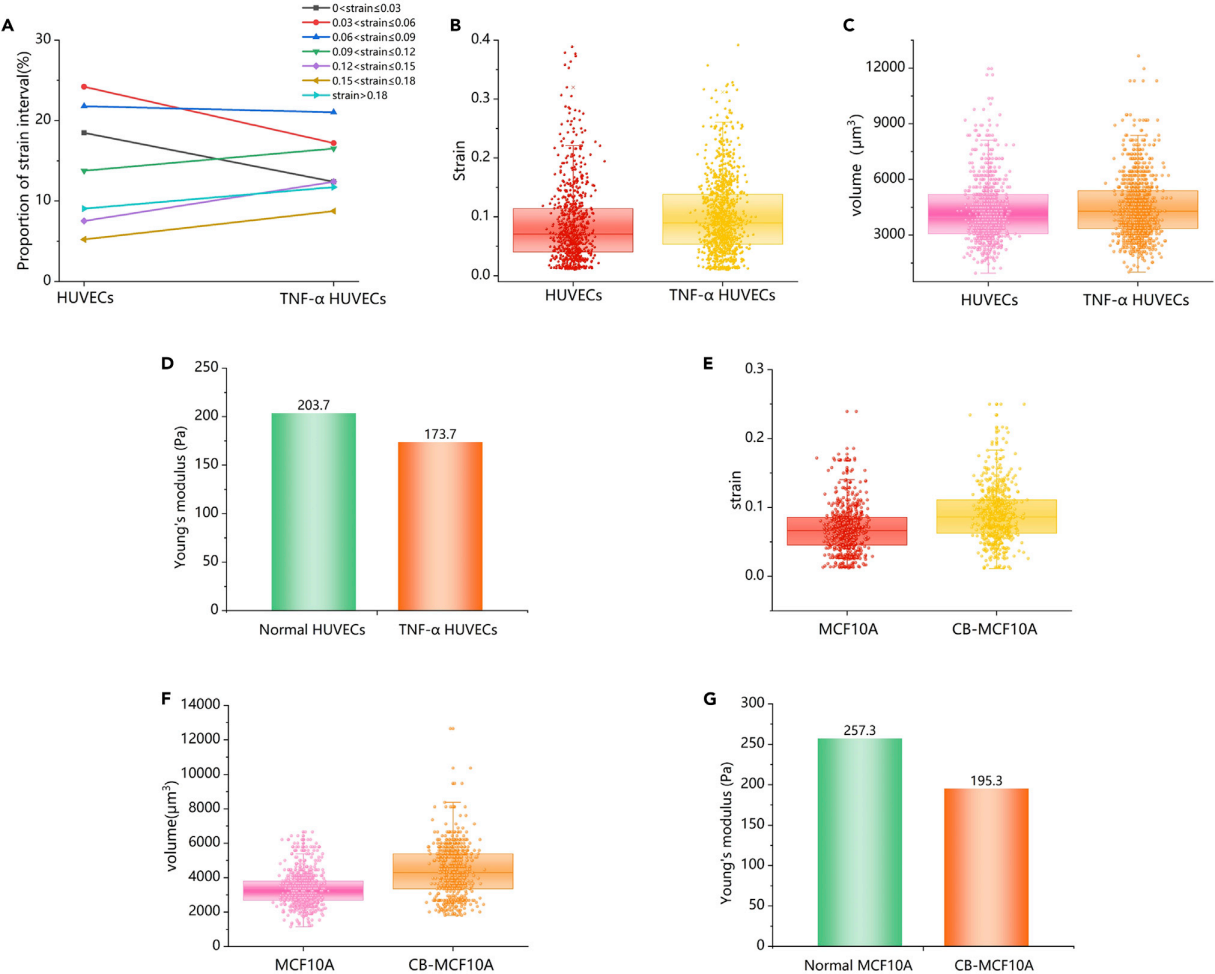
Comparison of mechanical characteristics and data analysis. Data are represented as median +/− confidence intervals (A) The ratio of the number of cells to the total number in different strain intervals in normal cells and drug-treated cells. (B and C) Columnar scatterplot of strain and volume of HU-VECs. (D) Changes in Young’s modulus before and after HUVECs treatment. (E and F) Columnar scatterplot of strain and volume of MCF-10A. (G) Changes in Young’s modulus before and after MCF-10A treatment.

The system mainly tested the data from normal HUVECs (n = 777) and TNF-α-treated HUVECs (n = 1033). Figure 6A shows the change in the proportion of strain interval between normal cells and drug-treated cells. The proportion of normal cells in the three intervals from 0 to 0.09 was significantly lower than that of drug-treated cells. By contrast, the proportion of normal cells in intervals greater than 0.09 was significantly higher than that of drug-treated cells. This proportion refers to the ratio of cells in a certain strain interval to the total number of measured cells. The number of “hard” cells with a relatively low strain value decreased after the drug treatment, whereas the number of "soft" cells with a relatively high strain value increased. Therefore, the cells tend to become soft after drug treatment. Figure 6B is a columnar scatterplot of cells before and after treatment. Although the strain value of cells was widely distributed, the average for drug-treated cells was increased, and the high-density area of the dots moved upward. This experimental phenomenon is difficult to observe in the case of low data volume. Hence, obtaining large quantities of data is statistically important. In addition, Young’s modulus was also calculated from stress-strain values (Figure 6D): the average strain value of HUVECs was increased from 0.0864 to 0.1013, and Young’s modulus was decreased from 203.7 Pa to 173.7 Pa.

The stiffness of TNF-α treated HUVECs was lower than that of normal HUVECs, and this finding is consistent with the description of Stroka et al., (2012). The increase in HUVEC volume after TNF-α treatment could explain the decrease in cell stiffness (Figure 6C). TNF-α is involved in the regulation of ion channels, which play an important role in cell volume regulation. The TNF-α treatment activates swelling-activated chloride channels in smooth muscle cells and increases the peak of intracellular Ca^2+^ concentrations in response to acetylcholine and bradykinin (Matsuda et al., 2010; White et al., 2006). Therefore, the increase in cell volume is accompanied by the influx of water and ions, resulting in the reduced stiffness of the cell.

To further verify the reliability of the system, we also measured different types of cells (MCF-10A). Normal MCF-10A (n = 701) and CB-treated MCF-10A (n = 615) were measured. CB is an inhibitor of actin polymerization, which can bind to intracellular microfilaments to cut the microfilaments and block the polymerization of actin at the end of the microfilaments. Figures 6E and 6G illustrate that the mechanical properties of MCF-10A changed significantly after CB treatment. The average strain value of MCF-10A was increased from 0.0684 to 0.0901 and the average Young’s modulus of MCF-10A was decreased from 257.3 to 195.3, thus indicating that the cells became softer. This result is also consistent with existing reports (Zhou et al., 2017). Figure 6F shows the change in cell volume after CB treatment. CB inhibits actin filaments resulting in changes in the cytoskeleton, which in turn leads to changes in cell morphology. Changes in cell volume may directly explain this.

## Conclusions

In response to the current problem of insufficient data for measuring the mechanical properties of cells using dielectrophoresis, this work proposes a novel automatic measurement method that combines a multifunctional dielectrophoretic microfluidic device and an automatic control scheme to obtain statistically significant results. The microfluidic device integrated with capture-stretch-release functions was designed to be compatible with automated methods. After capture and stretch were completed, the cell release operation was used to achieve multi-batch automated measurement of cell mechanical properties. The automatic control scheme including a deep learning-based cell detection algorithm and a hardware manipulation system was proposed to realize multi-batch cyclic measurement. A capture and release efficiency test utilizing PS beads was performed, and the findings showed that the capture efficiency was related to the size of the narrow mouth and the release efficiency was associated with the reverse flow rate. Finally, the optimal design was obtained. The selected capture narrow-mouth of 6 μm size and reverse flow rate of 150 μL/min resulted in capture efficiency of 84.17% and release efficiency of almost 100%, thereby meeting the needs of automated operation. Cell experiments were subsequently carried out by testing two different types of cells. Both results showed that the mechanical properties of cells were altered after drug treatment. This effect is biologically significant. Experimental results also confirmed that a large amount of cellular mechanical property data can be obtained automatically. The proposed unique automated high-throughput method shows a proven path to obtain statistically significant results for the measurement of cellular mechanics and is of great importance in the field of biology, medicine, and biomedical engineering.

## Limitations of the study

The proposed method greatly improves the efficiency, convenience, and accuracy of dielectrophoresis-based cell mechanical properties measurement, but the throughput is still insufficient. For a large number of cells to be tested, it still takes a long time to complete. In addition, although the microfluidic chip has been subjected to anti-adhesion treatment, there is still the possibility of adhesion and blocking the channel after a long time of measurement. Future study should further enhance the measurement efficiency, and utilize the materials with better anti-adhesion effect, as well as the anti-blocking design.

## STAR★Methods

### Key resources table

**Table:** 

REAGENT or RESOURCE	SOURCE	IDENTIFIER
**Chemicals, peptides, and recombinant proteins**		

Cytochalasin B	Procell	CAS:14,930-96-2
TNF-α	Procell	CAS:191732-72-6

**Experimental models: Cell lines**		

MCF-10A	Procell	CSTR:19375.09.3101HUMSCSP575
HUVEC	Procell	FH1122

**Software and algorithms**		

Python	Python Software Foundation	http://www.python.org
Numpy	Python Data Analysis Library	http://numpy.org
Serial	Python Data Analysis Library	http://serial.org
OpenCV	Python Data Analysis Library	http://opencv.org
YOLOv5	open source website	https://github.com/ultralytics/yolov5

### Resource availability

#### Lead contact

Further information and requests for resources should be directed to and will be fulfilled by the lead contact, Hao Yang (yhao@suda.edu.cn).

#### Materials availability

This study did not generate new unique reagents.

### Experimental model and subject details

#### Cell culture and sample preparation

HUVECs (Procell, China) and MCF-10A (Procell, China) were cultured in RPMI 1640 and DMEM medium with 10% fetal bovine serum (FBS) at 37°C in a 5% CO_2_ atmosphere, respectively. The culture medium was replaced every 2–3 days. When the cells grew to 80% density, they were washed, treated with trypsin, centrifuged, and resuspended for passage or subsequent experiments. In the experiments, isotonic buffer (0.3wt% glucose, 8.5wt% sucrose, appropriate amount of CaCl_2_ for conductivity regulation) was configured for long-term cell experiments. TNF-α was added to the culture medium of HUVECs to a concentration of 3 ng/mL to study its effect on cell mechanical properties, and the cells were cultured in a 6 mL medium containing TNF-α for 20 h to promote an inflammation response(Lee et al., 2011). CB was added to the culture medium of MCF-10A to a concentration of 3 μg/mL to study its effect on cell mechanical properties, and the cells were cultured in a 6 mL medium containing CB for 24 h. The TNF-α-treated HUVECs and CB-treated MCF-10A were extracted using the method for HUVECs and MCF-10A for the experiment analysis. The PS beads were stored at 4°C and diluted to produce a certain concentration of PS bead suspension for experimentation.

### Method details

#### Fabrication of the microfluidic device

The microfluidic device consisted of a polydimethylsiloxane (PDMS) microchannel and a glass sheet coated with ITO. SU-8 2015 was spin-coated with the first homogenization speed of 500 rpm for 10 s and then spin-coated with the second homogenization speed of 2000 rpm for 30 s to reach a 25 μm height. The mold was then fabricated on a Cr mask using photolithography techniques. PDMS microchannels were produced from the mold using Sylgard184 to mix the basic components and curing agent in a ratio of 10:1. A PDMS microchannel was formed after being heated at 85°C for 2 h. RZJ-304 positive photoresist was rotated at a primary speed of 800 rpm for 8 s and then rotated at a secondary speed of 3500 rpm for 30 s. A 5 μm-thick electrode structure protective layer was formed, and excess ITO was removed by hydrochloric acid etching. Finally, the photoresist was stripped with a stripping solution to obtain an ITO electrode. Under the action of oxygen plasma, the PDMS microchannel and the ITO electrode were aligned and bonded to form the final microfluidic device. The device was immersed in 0.1% (w/v) pluronic F-127 for 24 h to prevent cell adhesion.

#### Cell Young’s modulus characterization through DEP

DEP refers to the migration caused by induced dipole moment in a non-uniform electric field (Pohl 1951). The cell was assumed an ellipsoid model to evaluate the DEP force acting on it. The time-averaged DEP force can be described as follows (Gascoyne and Vykoukal 2002):

(Equation 2) FDEP=2πr3·εm·Re(fcm)·∇Erms2,

where *r* indicates the radius of the spherical cell, $\;\varepsilon_{m}$ denotes the permittivity of isotonic buffer, $E_{rms}$ is the root-mean-square value of the electric field strength, and $\;Re\left(f_{cm}\right)$ is the real part of the Clausius–Mossotti factor defined as:

(Equation 3) $$f_{cm}=\frac{\varepsilon_{p}^{*}-\varepsilon_{m}^{*}}{\varepsilon_{p}^{*}+2\varepsilon_{m}^{*}},$$

where $\varepsilon^{*}$ is complex permittivity; subscripts p and *m* represent particles and media, respectively, and are calculated by $\varepsilon^{*}=\text{ε}-j\sigma /\omega$; σ represents conductivity; and ω represents the frequency of the electric field. $Re\left(f_{cm}\right)$ can be changed from −0.5 to 1 according to the parameter. When $Re\left(f_{cm}\right)$ is positive, the polarized particles move to the place with the maximum electric field strength (*p*-DEP). When $Re\left(f_{cm}\right)$ is negative, the particles move away from the area of maximum electric field strength (n-DEP).

The stress generated by the DEP force acting on the cell can be expressed as follows(MacQueen et al., 2010):

(Equation 4) $$\sigma_{stress}=nRe\left(f_{cm}\right)\varepsilon_{m}E_{0}^{2},$$

(Equation 5) $$n=\frac{E}{r\frac{d\left|E\right|}{dx}},$$

where *n* is the geometric constant that can be obtained from the simulation, and $E_{0}$ is the electric field strength. The value of *n* was determined through COMSOL simulation.

Young’s modulus is defined as the ratio of stress to strain, the formula is as follows:

(Equation 6) $$\text{E}=\frac{\sigma }{\varepsilon }=\frac{nRe\left(f_{cm}\right)\varepsilon_{m}E_{0}^{2}}{\varepsilon }.$$

### Quantification and Statistical analysis

Statistical analysis and graph drawing are performed in the Origin software. The mean and SD are used to denote the center and range, respectively, and 6 sets of data are measured for each design (Figure 4A). The experiments measure normal HUVEV, TNF-α-treated HUVEC, normal MCF-10A, and CB-treated MCF-10A, and the numbers are 1033, 777, 701, 615, respectively. The center of the box in the scatterplot represents the median, and the range of the box in the chart represents the 75% confidence interval (Figure 6).

## Data Availability

- All data reported in this paper will be shared by the lead contact upon request. - This paper does not report original code. - Any additional information required to reanalyze the data reported in this paper is available from the lead contact upon request.

## Supplemental information

Video S1. Automated process for capture and release of microspheres, related to Figure 4

Video S2. Automated process of cell capture, stretch and release, related to Figure 5 and Figure 6
